# Haemolysis as a first sign of thromboembolic event and acute pump thrombosis in patients with the continuous-flow left ventricular assist device HeartMate II

**DOI:** 10.1007/s12471-015-0786-2

**Published:** 2015-12-21

**Authors:** S. Akin, O.I. Soliman, A.A. Constantinescu, F. Akca, O. Birim, R.T. van Domburg, O. Manintveld, K. Caliskan

**Affiliations:** 1 Thoraxcenter, Department of Cardiology, Unit Heart Failure, Heart Transplantation & Mechanical Circulatory Support, Erasmus Medical Center, ’s-Gravendijkwal 230, 3015 CE Rotterdam, The Netherlands; 2 Department of Intensive Care Unit, Erasmus Medical Center, Rotterdam, The Netherlands; 3 Cardialysis, Clinical Research Management & Core Laboratories, Rotterdam, The Netherlands; 4 Department of Cardiothoracic Surgery, Erasmus Medical Center, Rotterdam, The Netherlands

**Keywords:** Thromboembolic event, Pump thrombosis, Haemolysis, Left ventricular assist device (LVAD), HeartMate II

## Abstract

**Background:**

Despite advances in pump technology, thromboembolic events/acute pump thrombosis remain potentially life-threatening complications in patients with continuous-flow left ventricular assist devices (CF-LVAD). We sought to determine early signs of thromboembolic event/pump thrombosis in patients with CF-LVAD, which could lead to earlier intervention.

**Methods:**

We analysed all HeartMate II recipients (*n* = 40) in our centre between December 2006 and July 2013. Thromboembolic event/pump thrombosis was defined as a transient ischaemic attack (TIA), ischaemic cerebrovascular accident (CVA), or pump thrombosis.

**Results:**

During median LVAD support of 336 days [IQR: 182–808], 8 (20 %) patients developed a thromboembolic event/pump thrombosis (six TIA/CVA, two pump thromboses). At the time of the thromboembolic event/pump thrombosis, significantly higher pump power was seen compared with the no-thrombosis group (8.2 ± 3.0 vs. 6.4 ± 1.4 W, *p* = 0.02), as well as a trend towards a lower pulse index (4.1 ± 1.5 vs. 5.0 ± 1.0, *p* = 0.05) and a trend towards higher pump flow (5.7 ± 1.0 vs. 4.9 ± 1.9 L m, *p* = 0.06).

The thrombosis group had a more than fourfold higher lactate dehydrogenase (LDH) median 1548 [IQR: 754–2379] vs. 363 [IQR: 325–443] U/L, *p* = 0.0001). Bacterial (*n* = 4) or viral (*n* = 1) infection was present in 5 out of 8 patients. LDH > 735 U/L predicted thromboembolic events/pump thrombosis with a positive predictive value of 88 %.

**Conclusions:**

In patients with a CF-LVAD (HeartMate II), thromboembolic events and/or pump thrombosis are associated with symptoms and signs of acute haemolysis as manifested by a high LDH, elevated pump power and decreased pulse index, especially in the context of an infection.

## Introduction

Left ventricular assist devices (LVADs) have increasingly become part of the arsenal in the treatment of end-stage heart failure [1–3]. Despite advances in pump technology, thromboembolic events and acute pump thrombosis remain potentially life-threatening complications [4–6]. The clinical presentation varies from acute malfunction of the pump with heart failure, arrhythmias and/or to systemic thromboembolic events. The exact prevalence and aetiology of pump thrombosis is uncertain [7]. Rates of thromboembolic events, including ischaemic stroke and acute pump thrombosis, vary between 1 and 14 % among different studies of continuous flow (CF) LVADs with either axial or centrifugal flow [6, 8–15].

Recently, Starling et al. reported an increasing rate of Thoratec HeartMate II pump thrombosis, which was preceded by increasing lactate dehydrogenase (LDH), and was associated with substantial morbidity and mortality [16]. Other reports showed a strong association of haemolysis with increased pump power and with partial or complete LVAD thrombosis [17, 18]. The current guidelines of the International Society for Heart and Lung Transplantation (ISHLT) advise to follow up haemolysis as a sign of thrombosis [7]. Haemolysis in the presence of altered pump function should prompt admission for optimisation of anticoagulation and antiplatelet management and possible pump exchange [4]. However, there is no advice in the guidelines about detection of thrombosis. Clinically obvious haemolysis could be seen as dark urine, anaemia, jaundice, and/or as elevated LDH. Early detection of a thromboembolic event/pump thrombosis could help in the proper management of these LVAD patients.

As thromboembolic events and pump thrombosis are part of the same disease spectrum, we sought to analyse the determinants of thromboembolic events and/or pump thrombosis in the cohort of patients with a CF-LVAD implanted at our institution.

## Methods

Forty consecutive patients implanted with axial type continuous-flow HeartMate II LVADs (Thoratec Corporation, Pleasanton, California) in our institution, a tertiary referral centre for end-stage heart failure and heart transplantation, between December 2006 and July 2013, were included in this study.

### Data collection

All data from LVAD recipients were stored electronically in the hospital electronic patient records. According to Dutch law, informed consent was not required, since study-specific actions were not implemented. All data were readily available in the medical records of the patients and were obtained during routine treatment. Subsequently, data were processed anonymously. Data were retrospectively analysed for demographic, clinical and LVAD pump parameters. Clinical events such as signs of haemolysis, heart failure or infections were examined and confirmed independently by two cardiologists (SA, KC).

### Clinical and laboratory investigation

Clinical data, ECG, laboratory and echocardiography were collected every 2–3 months or more frequently according to the clinical need. Likewise, LVAD interrogation was performed regularly at every outpatient clinic visit by an LVAD technician. The last 12-lead ECGs before LVAD implantation and at follow-up/events were analysed including rhythm, QRS width and QTc interval. Blood samples were collected serially to assess parameters of haemolysis, kidney and liver function as well as inflammation (Table 1). The treating cardiologist made the choice of and changes in medications including heart failure and antiarrhythmic drugs. Twenty-five of 40 patients (63 %) already had an implantable cardioverter defibrillator according to the current guidelines [19].

**Table 1:** Baseline characteristics of all patients with or without thromboembolic events

	Total population (*n* = 40)	Thromboembolic event or pump thrombosis (*n* = 8)	No thromboembolic event or pump thrombosis (*n* = 32)	*p*-value
*Demographics*				
Age at implantation, years	46 [41–57]	56 [48–58]	45 [39–55]	0.12
Male gender	26 (65)	6 (75)	20 (63)	0.69
Weight, kg	71 ± 13	75 ± 12	70 ± 13	0.41
BSA, m^2^	1.87 ± 0.22	1.92 ± 0.19	1.86 ± 0.22	0.44
BMI, kg/m^2^	22.5 ± 3.0	23.3 ± 2.9	22.3 ± 3.0	0.44
*Aetiology*				
Non-ischaemic cardiomyopathy	23 (57)	4 (50)	19 (59)	0.70
Ischaemic cardiomyopathy	17 (43)	4 (50)	13 (41)	0.70
*Comorbidity*				
Diabetes mellitus	3 (8)	0	3 (9)	1.0
Hypertension	4 (10)	1 (13)	3 (9)	1.0
Previous cardiac surgery	3 (8)	2 (25)	1 (3)	0.10
Previous PCI	15 (38)	2 (25)	13 (41)	0.69
Previous TIA/CVA	2 (5)	0	2 (6)	1.0
*INTERMACS class*	2.4 ± 1.0	2.8 ± 1.3	2.3 ± 0.9	0.28
I	10 (25)	2 (25)	8 (25)	1.0
II	9 (23)	1 (12.5)	8 (25)	0.66
III	16 (40)	2 (25)	14 (44)	0.44
IV	5 (13)	3 (38)	2 (6)	0.05
Inotropic support	35 (87.5)	5 (63)	30 (94)	0.05
Extra-corporal circulatory support	9 (23)	2 (25)	7 (22)	1.0
Intra-aortic balloon pump	13 (33)	0	13 (41)	0.04
*LVAD parameters at discharge*				
Pump speed, rpm	9325 ± 516	9375 ± 225	9313 ± 568	0.76
Pump flow, L/m	4.9 ± 1.2	4.8 ± 1.0	5.0 ± 1.2	0.67
Pulse index	4.8 ± 0.9	4.9 ± 0.7	4.8 ± 0.9	0.92
Pump power, Watts	6.0 ± 1.3	6.0 ± 1.0	6.0 ± 1.3	0.94
*Electrocardiography*				
Atrial fibrillation	3 (8)	0	3 (9)	1.0
QRS duration, ms	146 ± 71	160 ± 54	143 ± 75	0.56
QTc, ms	462 ± 49	506 ± 35	451 ± 46	0.003
*Echocardiography*				
Left atrial dimensions, mm	48 ± 12	51 ± 9	47 ± 12	0.51
LV end-diastolic dimension, mm	67 ± 16	63 ± 14	66 ± 17	0.46
LV end-systolic dimension, mm	61 ± 16	63 ± 14	61 ± 17	0.76
*Baseline laboratory values*				
Lactate dehydrogenase, U/L	407 [321–849]	361 [277–455]	433 [333–1101]	0.21
NT-proBNP, pmol/L	1136 ± 1112	800 ± 471	1222 ± 1216	0.35
Total bilirubin, umol/L	24 ± 21	20 ± 14	25 ± 23	0.52
BUN, mmol/L	16 ± 10	23 ± 17	14 ± 7	0.03
Creatinine, umol/L	147 ± 89	143 ± 65	148 ± 95	0.89
CRP mg/L	56 ± 71	44 ± 54	59 ± 75	0.56
ALAT, U/L	314 ± 684	126 ± 227	361 ± 752	0.39
ASAT, U/L	297 ± 636	149 ± 268	334 ± 696	0.47
Albumin, g/L	30 ± 6	29 ± 4	30 ± 7	0.66
Haemoglobin, mmol/L	7.1 ± 1.2	7.5 ± 1.4	7.0 ± 1.1	0.26
Haematocrit l/l	0.35 ± 0.06	0.38 ± 0.08	0.34 ± 0.06	0.19
WBC count, 1000/mm^3^	10.1 ± 5.4	9.3 ± 2.7	10.3 ± 5.9	0.63
Platelet count, 1000/mm^3^	207 ± 89	250 ± 97	196 ± 85	0.13

Categorical variables are presented as frequencies and percentages. Continuous variables are presented as mean ± standard deviation or median [IQR (interquartile range 25th, 75th percentile)].

*IQR* interquartile range, *BSA* body surface area, *BMI* body mass index, *PCI* percutaneous coronary intervention, *TIA* transient ischaemic attack, *CVA* ischaemic cerebrovascular accident, Interagency Registry for Mechanically Assisted Circulatory Support (INTERMACS), *LVAD* left ventricular assist device, *rpm* revolutions per minute, *NT-pro-BNP* N-terminal of the prohormone brain natriuretic peptide, *BUN* blood urea nitrogen, *CRP* C-reactive protein, *ALT* alanine aminotransferase, *AST* aspartate aminotransferase, *WBC* white blood cell.

### Antithrombotic therapy

According to the ISHLT guidelines, postoperative anticoagulation started after LVAD implantation and completed postoperative haemostasis [7]. On postoperative day 1–2, intravenous heparin was started if there was no evidence of bleeding. On day 2–5, after removal of the chest tubes, aspirin 80 mg daily and vitamin K antagonists were started with a target international normalised ratio (INR) of 2.0–2.5. In case of a suspected thromboembolic event/pump thrombosis, intravenous heparin was started along with clopidogrel 75 mg/day. The target INR was increased to 2.5–3.5 or 3.0–4.0 in case of asymptomatic (laboratory only) signs of haemolysis versus thromboembolic event/pump thrombosis, respectively. In case of acute pump thrombosis, thrombolytic therapy (alteplase: bolus 15 mg in 1–2 min, followed by 0.75 mg/kg (max. 50 mg) continuous infusion in 90 min, and 0.5 mg/kg (max. 35 mg) continuous infusion in the second 90 min) was given.

### Outcome definitions

Pump thrombosis was defined as signs and symptoms of otherwise unexplained heart failure with signs of LVAD dysfunction and haemolysis, thromboembolic events as cerebrovascular accident (CVA) or transient ischaemic attack (TIA), as confirmed by a neurologist. Haemolysis was diagnosed according to the ISHLT guidelines and the Interagency Registry of Mechanically Assisted Circulatory Support (INTERMACS) on analysis of pump thrombosis [7, 20]. Laboratory and clinical diagnosis of LVAD haemolysis and thrombosis were considered according to these ISHLT guidelines and the INTERMACS registry. In the ISHLT guidelines, screening for haemolysis is indicated in the setting of an unexpected drop in the haemoglobin or haematocrit level along with other clinical signs of haemolysis, such as haematuria. Screening for haemolysis with serum LDH, plasma free haemoglobin in addition to the haemoglobin or haematocrit level is recommended [7]. The INTERMACS recently specified a new definition, accepted by the US Food and Drug Administration and industry, indicating a lower threshold of biochemical markers of haemolysis. They used a cut-off value of serum free haemoglobin > 40 mg/dl in association with clinical signs of haemolysis beyond 72 h post-implantation to define haemolysis [7].

### Statistical analysis

Categorical variables are presented as frequencies and percentages. Continuous variables are presented as mean ± standard deviation or median (interquartile range 25th, 75th percentile). Continuous variables were compared using paired or independent t-test, Mann–Whitney U-test or Wilcoxon’s test when appropriate. When comparing frequencies, the Chi-square or Fisher’s exact test was used, where applicable. Cumulative Kaplan–Meier survival curves were constructed for each outcome variable. All tests were two-tailed and *p*-values less than 0.05 were considered statistically significant. All *p*-values between 0.05 and 0.10 were considered to be a statistical trend. Multivariate analysis was not done due to the too low number of events and small population.

## Results

In our single-centre LVAD cohort of bridge-to-transplant patients, we found clinical features and other factors associated with thromboembolic events and acute pump thrombosis in 8 of the 40 patients. One out of 5 patients on LVAD support with HeartMate II developed this catastrophic complication during a median follow-up of approximately 18 months. Thromboembolic events/pump thrombosis occurred at a minimum of 34 days and a maximal of 649 days after implantation. Demographic, clinical, laboratory, pump and echocardiography characteristics of the thrombosis and no-thrombosis groups at baseline and at follow-up are listed in Table 1 and 2, respectively. The patients were divided into two groups with and without a thromboembolic event and/or pump thrombosis. The baseline data are from the pre-implant period, the laboratory values from the day before the operation. In all patients, the LVAD was implanted as a bridge to transplant. However, in three patients LVAD support ended in destination therapy (severe CVAs in two and malignancy in one). In one patient, the LVAD could be explanted due to cardiac recovery. All patients were followed for a median of 336 (IQR 182–808) days. No patients were lost to follow-up.

**Table 2:** Comparison of outcome in patients with and without acute pump thrombosis/thromboembolic events at the time of the event or last follow-up

	Total population (*n* = 40)	Thromboembolic event or pump thrombosis (*n* = 8)	No thromboembolic event or pump thrombosis (*n* = 32)	*p*-value
*Follow-up, days*	336 [182–808]	549 [269–856]	297 [152–806]	0.39
Death	8 (20)	0 (0)	8 (25)	0.17
Heart transplantation	18 (45)	4 (38)	14 (44)	1.0
On-going support	12 (30)	3 (38)	9 (28)	0.68
LVAD explantation	1 (3)	1 (13)	0 (0)	0.20
*LVAD parameters* ^a^				
Pump speed, rpm	9245 ± 364	9200 ± 283	9256 ± 384	0.70
Pump flow, L/m	5.1 ± 1.0	5.7 ± 1.0	4.9 ± 0.9	0.06
Pulse index	4.8 ± 1.2	4.1 ± 1.5	5.0 ± 1.0	0.05
Pump power, Watts	6.8 ± 1.9	8.2 ± 3.0	6.4 ± 1.4	0.02
*Clinical haemolysis parameters*				
Macroscopic hemoglobinuria	7 (18)	4 (50)	3 (9)	0.02
LDH levels > 735 U/L	20 (50)	7 (88)	13 (41)	0.04
Free Hb (> 6 indicates haemolysis)	15 ± 34	33 ± 58	10 ± 22	0.31
Infection at the time of TE/PT	15 (38)	5 (63)	10 (31)	0.13
Viral	5 (13)	1 (13)	4 (13)	1.0
Bacterial	10 (25)	4 (50)	6 (19)	0.07
*Readmissions*				
Surgery for driveline fracture	6 (15)	1 (13)	5 (16)	1.0
Re-admission due to HF	8 (20)	2 (25)	6 (19)	0.65
*Medications at TE/PT or latest follow-up*				
Vitamin K antagonist	33 (83)	8 (100)	25 (78)	0.31
Aspirin	30 (75)	7 (88)	23 (72)	0.65
Clopidogrel	4 (10)	2 (25)	2 (6)	0.17
*Electrocardiography*				
Atrial fibrillation	6 (15)	1 (13)	5 (16)	0.82
QRS duration, ms at event	148 ± 47	161 ± 45	145 ± 48	0.40
QTc, ms at event	463 ± 75	535 ± 79	445 ± 63	0.001
*Echocardiography*				
Grade aortic regurgitation	1.0 ± 0.9	1.1 ± 1.0	1.0 ± 0,9	0.66
Grade mitral regurgitation	1.2 ± 1.1	1.3 ± 0.9	1.2 ± 1.2	0.89
LV end-diastolic dimension, mm	57 ± 15	61 ± 14	56 ± 16	0.42
LV end-systolic dimension, mm	50 ± 14	52 ± 13	50 ± 15	0.73
Left atrial dimensions, mm	38 ± 11	38 ± 9	38 ± 11	1.0
*Laboratory findings*				
LDH, U/L	382 [331–591]	1548 [754–2379]	363 [325–443]	< 0.0001
NT-proBNP, pmol/L	473 ± 890	915 ± 1551	342 ± 558	0.11
Total bilirubin, umol/L	38 ± 79	24 ± 13	41 ± 88	0.60
BUN, mmol/L	10 ± 7	12 ± 9	10 ± 7	0.56
Creatinine, umol/L	136 ± 166	152 ± 148	132 ± 172	0.77
CRP, mg/L	70 ± 91	84 ± 92	67 ± 91	0.64
ALAT, U/L	82 ± 159	162 ± 226	62 ± 135	0.11
ASAT, U/L	121 ± 203	185 ± 156	106 ± 212	0.33
Albumin, g/L	39 ± 9	40 ± 8	39 ± 10	0.77
INR	2.3 ± 1.1	2.4 ± 0.9	2.3 ± 1.1	0.89
Haemoglobin, mmol/L	6.7 ± 1.8	6.0 ± 1.7	6.9 ± 1.8	0.19
Haematocrit, l/L	0.33 ± 0.09	0.31 ± 0.07	0.34 ± 0.09	0.41
WBC count, 1000/mm^3^	10 ± 6	10 ± 6	10 ± 6	0.99
Platelet count, 1000/mm^3^	198 ± 84	237 ± 71	188 ± 84	0.14

Categorical variables are presented as frequencies and percentages. Continuous variables are presented as mean ± standard deviation or median [IQR (interquartile range 25th, 75th percentile)].

*p* value < 0.05 is significant. *p* value 0.05–0.10 is called tendency.

*IQR* Interquartile range. *LVAD* left ventricular assist device, *rpm* revolutions per minute, *LDH* lactate dehydrogenase, *free Hb* free haemoglobin, *TE* thromboembolic event, *PT* pump thrombosis, *HF* heart failure, *LV* left ventricle, *NT-pro-BNP* N-terminal of the prohormone brain natriuretic peptide, *BUN* blood urea nitrogen, *CRP* C-reactive protein, *ALAT* alanine aminotransferase, *ASAT* aspartate aminotransferase, *INR* international normalised ratio, *WBC* white blood cell.

^a^LVAD parameters at event or latest follow-up; Values presented as mean (SD), median (interquartile range), or *n* (%).

**Fig. 1:**
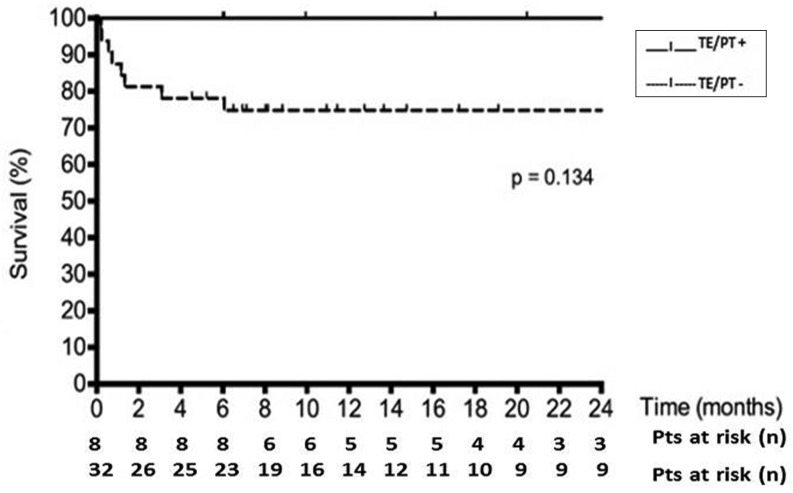
Kaplan-Meyer curve for survival during LVAD support for the thrombosis group (TE/PT+) and the no-thrombosis group (TE/PT−). Patients are censored at heart transplantation and LVAD explantation

**Fig. 2:**
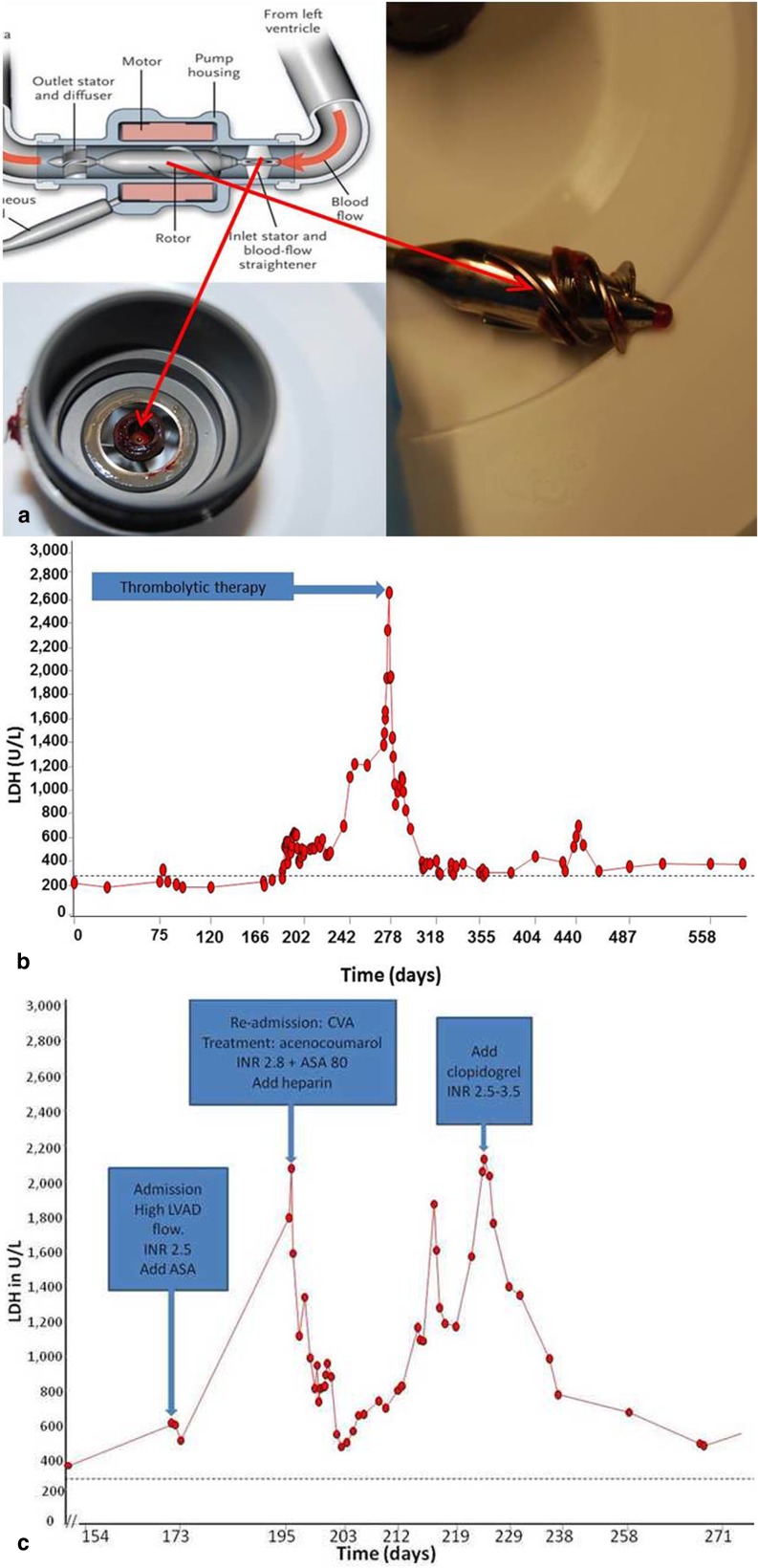
**a** Explanted pump inlet rotor in a 54-year-old male (patient no. 9 in Table 3) with acute pump thrombosis. Due to acute pump thrombosis, the patient had acute left- and right-sided heart failure with signs of severe haemolysis and acute renal failure. Macroscopic fresh white and red old thrombus is shown on the rotor, as confirmed by the manufacturer. **b** LDH course of the 57-year-old male (patient no. 7 in Table 3) presenting with acute pump thrombosis successfully treated with recombinant tissue-plasminogen activator (rt-PA). This patient had several episodes of an abrupt peak of LDH during therapeutic INRs associated with relapsing urinary tract infections (Citrobacter freundii). At the highest LDH peak he developed acute pump thrombosis, which was treated with thrombolytic therapy (alteplase). *Dashed line* = upper limit of normal value LDH. **c** Time course of serum LDH (U/L) in a 37-year-old woman (patient no. 5 in Table 3), 6 months on LVAD support, admitted with a ischaemic cerebrovascular event and response to various therapeutic interventions. *CVA* cerebrovascular accident, *INR* international normalised ratio, *ASA* acetylsalicylic acid, *iv* intravenous. *Dashed line* = upper limit of normal value LDH

At baseline, an intra-aortic balloon pump was used significantly more often in the thrombosis group, and this group had a higher blood urea nitrogen. Furthermore, the thrombosis group showed a trend towards INTERMACS class IV (*p* = 0.05) and less inotropic use (*p* = 0.05). At the last follow-up (July 2013), eight (20 %) patients had one or more thromboembolic events or pump thrombosis at median follow-up of 549 [269–856] days: TIA in 4 patients, ischaemic CVA in 3 patients and acute pump thrombosis in 2 patients. There was no difference in survival between the groups censored for heart transplantation or LVAD explantation (*p* = 0.13, Fig. 1). One patient with pump thrombosis was treated with acute pump replacement (Table 3 and Fig. 2a) and one patient underwent successful thrombolysis (Fig. 2b). Four patients in the no-thrombosis group had a non-ischaemic CVA, one due to an air embolism and three due to intracerebral bleeding. In these patients, a neurologist ruled out an ischaemic stroke by CT scan. At the time of the thromboembolic event/pump thrombosis, higher pump power was seen in thrombosis group compared with the no-thrombosis group (8.2 ± 3.0 vs. 6.4 ± 1.4 W, *p* = 0.02), as well as a trend towards a lower pulse index (4.1 ± 1.5 vs. 5.0 ± 1.0 *p* = 0.05) and a trend towards higher pump flow (5.7 ± 1.0 vs. 4.9 ± 1.9 L m *p* = 0.06) Macroscopic haemoglobinuria was seen in 4 of 8 patients of the thrombosis group and 3 of 32 patients of no-thrombosis group (50 vs. 9 %, *p* = 0.02). The patients in the thrombosis group with macroscopic haemoglobinuria had concomitant infections; at that time they had a therapeutic INR or activated partial thromboplastin time. They were all empirically treated with intravenous heparin and clopidogrel. None developed a thromboembolic event or pump thrombosis. The presence of a thrombus in the pump was confirmed at explantation by the thoracic surgeon and manufacturer in 4 of the 8 patients.

**Table 3:** Detailed overview of the eight patients with thromboembolic events during follow-up

Patient no.	1	2	3	4	5	6	7	8
Age (years)	46	49	57	64	37	59	57	54
Sex	M	M	M	M	F	F	M	M
Aetiology heart failure	CMP	CMP	IHD	IHD	CMP	IHD	CMP	IHD
INTERMACS class	4	4	2	3	1	1	3	4
Total support time (days)	1603	614	1057	789	483	182	298	180
Time to event (days)	631	175	649	34	195	71	89	49
Event	TIA	TIA	CVA	TIA	CVA	TIA + CVA	Pump thrombosis	Pump thrombosis
Infection at the time of the event	Viral upper airway infection	None	Sepsis e.c.i	None	None	Urinary tract infection	Bacterial prostatitis	Urinary tract infection
Culture	None	None	*Staphyl. species (CNS)*	None	None	*Enterococcus faecalis*	*Citrobacter freundi*	*Morganella morganii*
Treatment at the time of event	ASA/OAC	ASA/OAC	ASA/OAC	OAC^a^	ASA^b^/OAC	ASA/OAC	ASA/OAC	ASA/OAC
NT-proBNP (pmol/L)	85	48	339	398	65	233	4539	1611
INR	2.2	2.8	2.4	1.4	2.2	4.0	2.1	2.5
Macroscopic haematuria	No	No	No	No	Yes	Yes	Yes	Yes
Free-Hb	1	9	8	3	50	1	21	172
Peak LDH (U/L)	422	745	2286	757	2131	965	2658	3532
Target INR	3–4	2.5–3.5	2.5–3.5	2.5–3.5	Clopidogrel + 2.5–3.5	Clopidogrel + 2.5–3.5	Clopidogrel + 2.5–3.5 + alteplase	Urgent pump exchange
Success of treatment	Yes	Yes	Yes	Yes	Yes	Yes	Partly	No
Outcome	HTX	HTX	LVAD DT	HTX	Successfully explanted	HTX: death	Semi urgent HTX	Urgent HTX

*CMP* cardiomyopathy, *IHD* ischaemic heart disease, *TIA* transient ischaemic attack, *CVA* ischaemic cerebrovascular attack; e.c.i (e causa ignota), *CNS* coagulase-negative staphylococci, *ASA* aspirin, *OAC* oral anticoagulation, *INR* international normalised ratio, *Hb* haemoglobin, *LDH* lactate dehydrogenase, *HTX* heart transplantation, *LVAD* left ventricular assist device, *DT* destination therapy.

^a^No aspirin due to active duodenal ulcer.

^b^Aspirin started late (5 months post LVAD) due the peri-hepatic haematoma.

Furthermore, the thrombosis group had more than fourfold higher lactate dehydrogenase levels (median LDH: 1548 [IQR: 754–2379] vs. 363 [IQR: 325–443] U/L, *p* < 0.0001). In the thrombosis group, infection was associated with a more than threefold increase of LDH with or without clinical signs of TIA/CVA or pump thrombosis. In 63 % (vs. 31 % in the no-thrombosis group, *p* = 0.13) of the patients presenting with thromboembolism/pump thrombosis, an infection (bacterial in 4 and viral in 1 of the 8 patients) was confirmed. At baseline and at follow-up, there was a significantly longer corrected-QT interval in the thrombosis group.

The sensitivity and specificity of LDH as a marker of haemolysis (cut-off value three times the upper limit of normal (LDH > 735 U/L) were 88 and 97 %, respectively, with the positive and negative predictive value being 88 vs. 97 %, respectively.

## Discussion

In our single-centre LVAD cohort of bridge-to-transplant patients, we studied the clinical features and associated factors in patients with thromboembolic events and acute pump thrombosis. One out of 5 patients on LVAD support with a HeartMate II developed a thromboembolic event/pump thrombosis during a median follow-up of approximately 18 months. Infection was confirmed at the time of the event in two-thirds of these patients. Elevated pump power and macroscopic haemoglobinuria predicted thromboembolic events/pump thrombosis, but LDH more than three times the upper level of normal was the best biochemical parameter in predicting and guiding the management of LVAD-related thromboembolic events and/or acute pump thrombosis, with a sensitivity of 88 % and specificity of 97 %.

### Thromboembolic events in continuous-flow LVADs

CF-LVADs have been increasingly used in the last decade as a bridge to transplantation [21]. Thromboembolic events/pump thrombosis remain one the most feared common complications in CF-LVAD patients, although bleeding results in the most morbidity and mortality [22–23]. The anticoagulation strategy of Whitson et al. [6] has been nuanced because it requires a delicate balance of adequate anticoagulation to minimise thrombotic complications but not so excessive that it will cause bleeding complications (e.g., gastrointestinal or neurological bleeding). What further complicates the clinical picture is the inherent haematological effects of CF-LVADs [24–25]. In our cohort there were 23/40 cases of early post-implantation bleeding, 21 of which resulted in early cardiac tamponade. In their analysis in 2008, John et al. [12] found a low thromboembolic risk (4.4 %) in HeartMate II patients even with less stringent requirements for anticoagulation, which was confirmed by Menon et al. in 2012 [13]. From 2013, there has been an increase in thromboembolic events/pump thrombosis in HeartMate II patients to 13.4 %, according to Whitson et al. [6]. In our report, 5 of the 40 patients (12.5 %) had a CVA or pump thrombosis, if TIAs were excluded due their mild clinical sequelae.

### Acute pump thrombosis

Acute pump thrombosis is a life-threatening condition and its optimal management requires early intervention. Detection of the earliest signs of pump thrombosis could lead to successful thrombolysis of a soft developing thrombus [26]. Uriel et al. examined 177 LVAD patients of whom 19 (11 %) developed acute pump thrombosis, whereby all underwent pump exchange; the recurrence rate was 1 %. In one-third of the patients, inadequate anticoagulation was found due to withholding or cessation of anticoagulation [15]. Thrombolysis could also be used with varying effect, as in our cases in Fig. 2a, b and c [11, 27]. In our experience, the use of clopidogrel on top of aspirin and coumarins in optimising anticoagulation seems effective and safe, but the efficacy of antiplatelet therapy using clopidogrel in one study was not sufficient in more than 50 % of the patients [28].

### Haemolysis

In a multicentre analysis it was recently demonstrated that haemolysis causes long-term negative effects in the long-term course of LVAD support [29]. In 7 % of their 115 patients with a HeartMate II, Hasin et al. found signs of haemolysis presenting with very high LDHs (more than six times normal) which after intensifying the anticoagulation therapy decreased to baseline within 2 weeks [11]. However, recurrent haemolysis was very common: 75 % over 1–7 months [11]. A recent analysis of the INTERMACS Registry of 4850 patients frequently found a mean time to event of 7.4 months and a cumulative incidence of 9 % at 2 years [29]. In another cohort of 20 consecutive cases of pump thrombosis, haematological markers, including LDH, plasma free haemoglobin and creatinine, were the only reliable sign of LVAD thrombosis, as opposed to echocardiographic or pump parameters.[30] Along with the emerging association of LDH with thromboembolism in patients with HeartMate II, there is also a growing association with acute infections [31], probably due to increased hypercoagulability [15, 31]. We can confirm this in our cohort, where there seems to be a correlation between infection and thrombosis in LVAD. LDH seems to be a very powerful parameter in predicting serious thromboembolic adverse events in HeartMate II patients.

Interestingly, the QTc was longer in the thrombosis group compared with the no-thrombosis group both at baseline and at latest follow-up. It is known that QTc is a very crucial prognostic parameter in end-stage heart failure and we see here an association with the development of thrombosis [32]. To our knowledge there are no reports on LVAD studies that have described this before. Further studies are needed to analyse these novel findings.

### Study limitations

This study has several limitations, which should be taken into account in the final interpretation of the data. The design is a retrospective study, the number of cases is very limited, and there was no routine follow-up and analysis of eventual hypercoagulability and/or antiplatelet drugs resistance. Also, our findings were restricted to HeartMate II and may not be applicable to patients supported with other types of CF-LVADs.

## Conclusion

In patients with CF-LVAD (HeartMate II), thromboembolic events and/or pump thrombosis are associated with symptoms and signs of acute haemolysis as manifested by high LDH, elevated pump power and decreased pulse index, especially in the context of an infection. These symptoms and signs could help in the early diagnosis and timely intensification of antithrombotic and/or antiplatelet therapy to prevent acute pump thrombosis and thromboembolic events or the need for pump replacement.

### Funding

None.
